# Supplementary material for: Self-assembled cellulosic superstructures with unanticipated high quantum yields
Source: Nat Commun. 2025 Dec 10;17:176. doi: 10.1038/s41467-025-66277-8 (PMC12780042; doi:10.1038/s41467-025-66277-8)
Supplement: Supplementary file 1 — Supplementary Information [file 41467_2025_66277_MOESM1_ESM.pdf]

Supplementary Information

Self-assembled cellulosic superstructures with unanticipated high quantum yields

Cheng Li,[1] Zhen Lang,[1] Jade Poisson,[1] Wenbo Chen,[1] Caoxing Huang,[1,2*] Evgeny Nimerovsky,[3] Philipp Vana,[4,5] Kai Zhang[1,5*]

[1] Sustainable Materials and Chemistry, Department of Wood Technology and Wood-based Composites, University of Göttingen, Göttingen 37077, Germany

[2] Co-Innovation Center for Efficient Processing and Utilization of Forest Resources, College of Chemical Engineering, Nanjing Forestry University, Nanjing 210037, China

[3] Department of NMR-Based Structural Biology, Max Planck Institute for Multidisciplinary Sciences, Göttingen 37077, Germany

[4] Institute of Physical Chemistry, University of Göttingen, Göttingen 37077, Germany

[5] Wöhler Research Institute for Sustainable Chemistry (WISCh), University of Göttingen, Göttingen 37077, Germany

Corresponding authors:

Caoxing Huang - Email: hcx@njfu.edu.cn

Kai Zhang - Email: kai.zhang@uni-goettingen.de

This file includes:

Supplementary Figs. 1−20

Supplementary Discussion

Supplementary Note 1

Supplementary Tables 1−3

Supplementary References 1−6

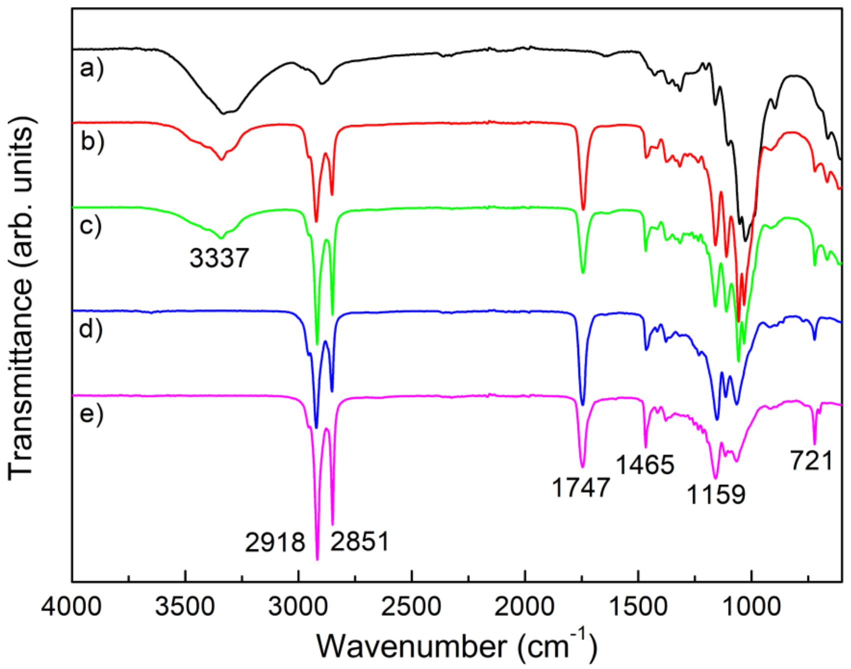

Supplementary Fig. 1. FTIR spectra of a) MCC, b) CNC-C12, c) CNC-C18, d) CLE, and e) CSE.

A comparative analysis of the FTIR spectra of MCC and its esterified derivatives (CSE, CLE, CNC-C18, and CNC-C12) reveals key structural changes. The complete disappearance of the hydroxyl stretching vibration peak at 3337 cm^{-1} in CSE and a sharp reduction in its intensity for CNC-C18/CNC-C12 compared to MCC confirm successful surface esterification. Concurrently, the asymmetric and symmetric C-H stretching vibrations of aliphatic chains (observed at 2918 cm^{-1} and 2851 cm^{-1}, respectively) exhibit significantly enhanced intensities in all modified samples relative to unmodified MCC. New absorption bands emerge at 1747 cm^{-1} (C=O stretching) and 1465 cm^{-1} (C-H deformation) in the spectra of esterified derivatives, providing further evidence of the introduction of aliphatic ester groups. Additionally, the presence of peaks at 1159 cm^{-1} (C-C stretching) and 721 cm^{-1} (C-C rocking) aligns with the characteristic vibrations of long aliphatic chains, corroborating the structural modifications induced by esterification.

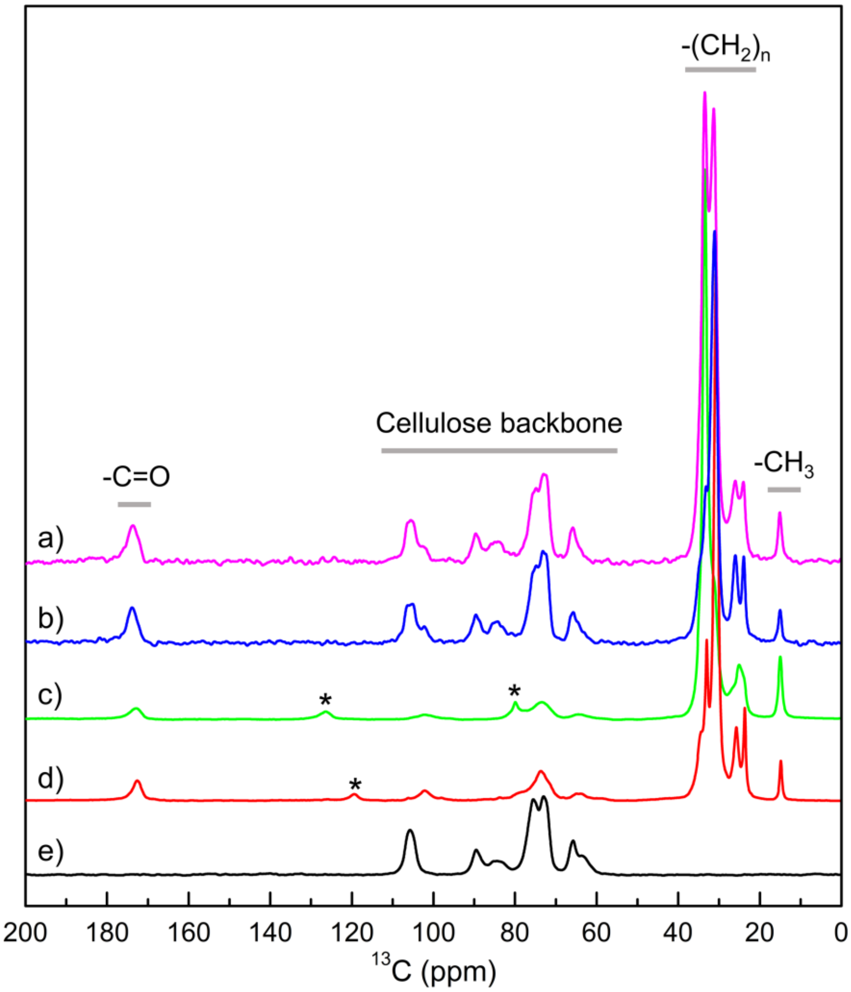

Supplementary Fig. 2. Solid-state ^{13}C NMR spectra of a) CNC-C18, b) CNC-C12, c) CSE, d) CLE, and e) MCC. The stars label the spinning sidebands.

Solid-state ^{13}C NMR spectroscopy further confirms the successful chemical modification. The spectral signals observed between 55 and 110 ppm correspond to carbons within the anhydroglucose units of the cellulose backbone. Critically, the distinct chemical shift at 173 ppm is assigned to the carbonyl carbon of the newly formed ester linkage, providing direct evidence of esterification. Additionally, the resonance signals in the 40–10 ppm region arise from the aliphatic carbons of the grafted stearoyl and lauroyl chains, confirming the incorporation of these hydrophobic groups into the cellulose matrix.

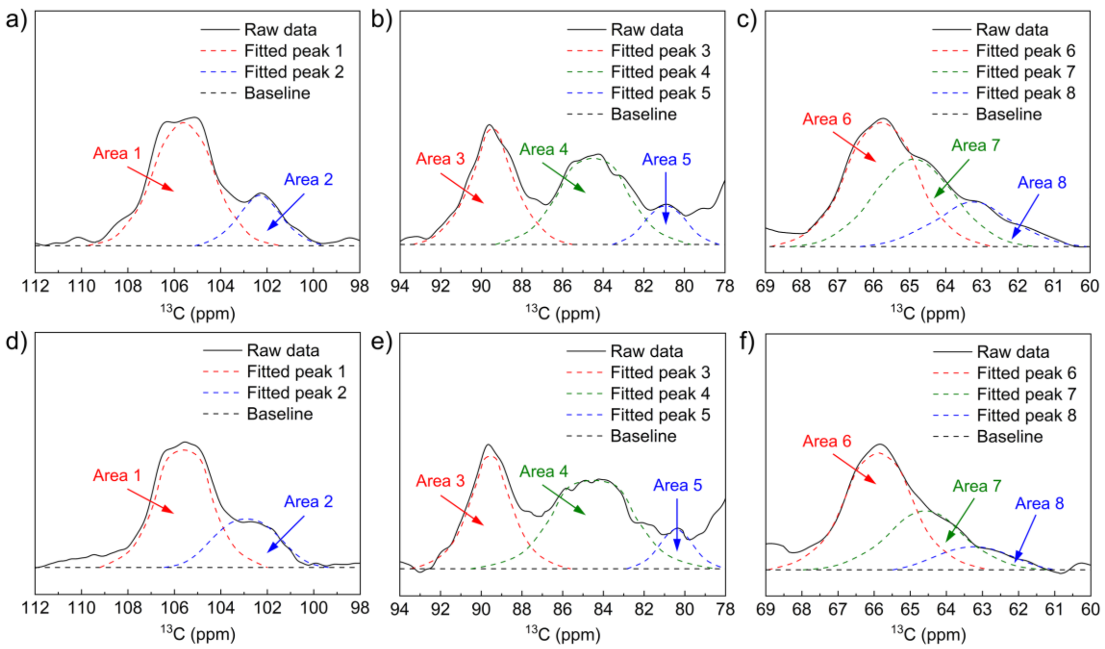

Supplementary Fig. 3. Solid-state ^{13}C NMR spectra of CNC-C12 showing the peak regions of a) C1, b) C4, c) C6. Solid-state ^{13}C NMR spectra of CNC-C18 showing the peak regions of d) C1, e) C4, f) C6. DS_2 = Area 2 / (Area 1 + Area 2), DS_3 = Area 5 / (Area 3 + Area 4 + Area 5), DS_6 = Area 7 / (Area 6 + Area 7 + Area 8).

The degree of substitution (DS) for C2 (DS_2) and C3 (DS_3) was determined from the peak splitting (upfield shift) of the adjacent carbons (C1 and C4), whereas the DS for C6 (DS_6) was calculated from the ratio of peaks corresponding to unsubstituted and substituted hydroxyl groups.[1] The results can be found in Supplementary Table 1.

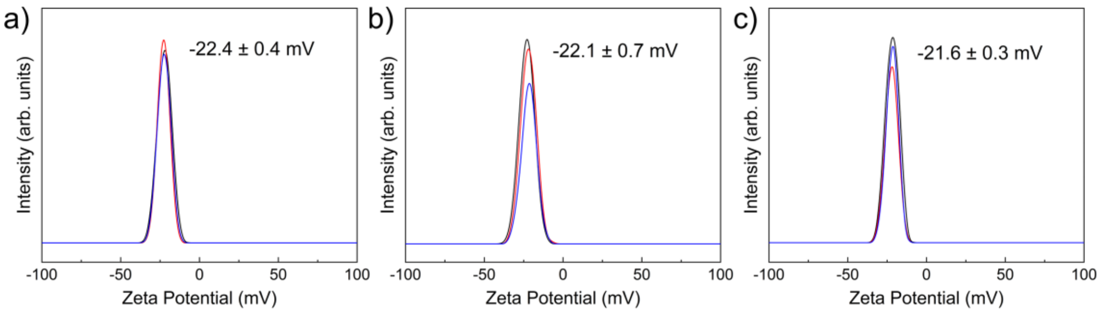

Supplementary Fig. 4. Zeta potential results of a) CNC, b) CNC-C12, and c) CNC-C18.

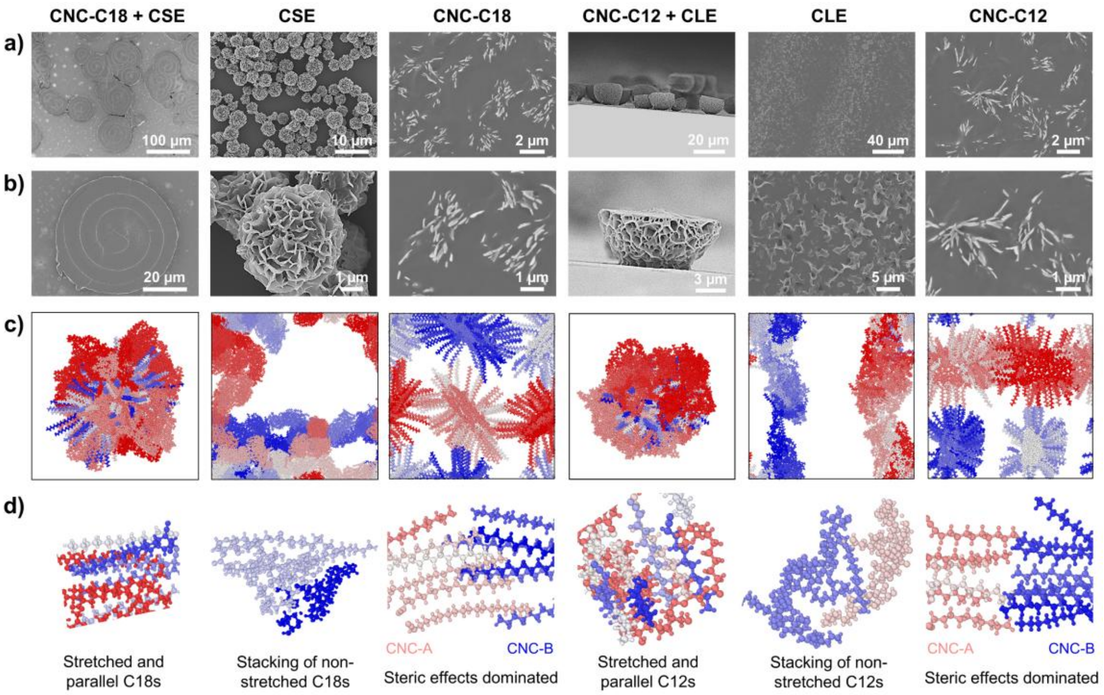

Supplementary Fig. 5. Morphologies of the superstructures and conformations of the hydrophobic chains for the six self- and co-assembly systems (labeled above). a,b) Scanning electron microscopy (SEM) images of self- and co-assembled superstructures from CNC-C18+CSE, CSE, CNC-C18, CNC-C12+CLE, CLE, and CNC-C12 systems, after the evaporation of solvents (tetrahydrofuran). c) Molecular dynamics (MD)-simulated equilibrium structures of the six systems corresponding to those in a,b). Molecules in the snapshots are color-coded by molecular identification (ID) to distinguish individual molecules. d) Partial enlargement of c), showing only the stearoyl and lauroyl groups.

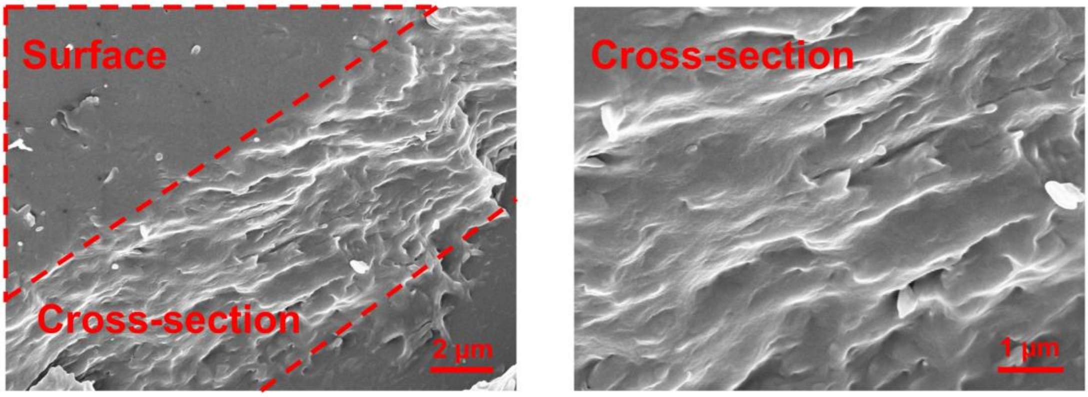

Supplementary Fig. 6. SEM images of the cross-section view of self-assembled MHs from CNC-C18+CSE. The images were captured using an inclined specimen holder

(45°).

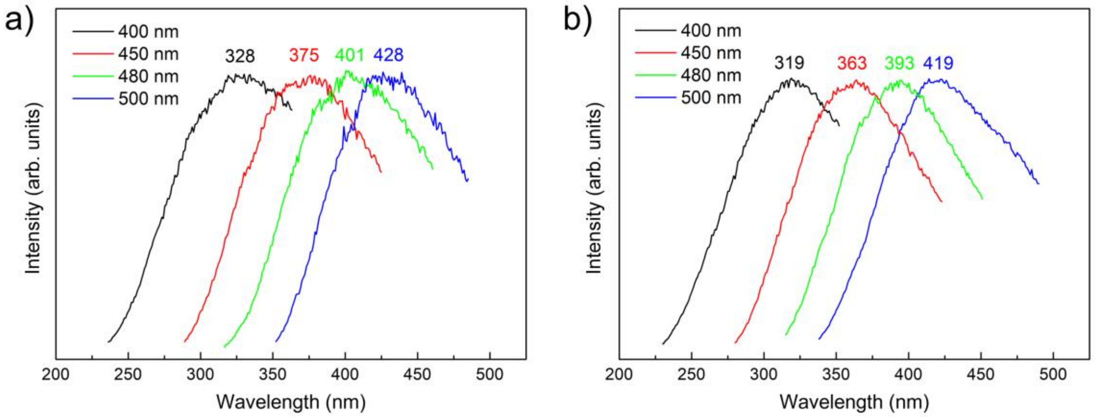

Supplementary Fig. 7. Normalized solid-state excitation spectra of a) MHs and b) bowl-shaped microparticles (BSMPs) at different emission wavelengths.

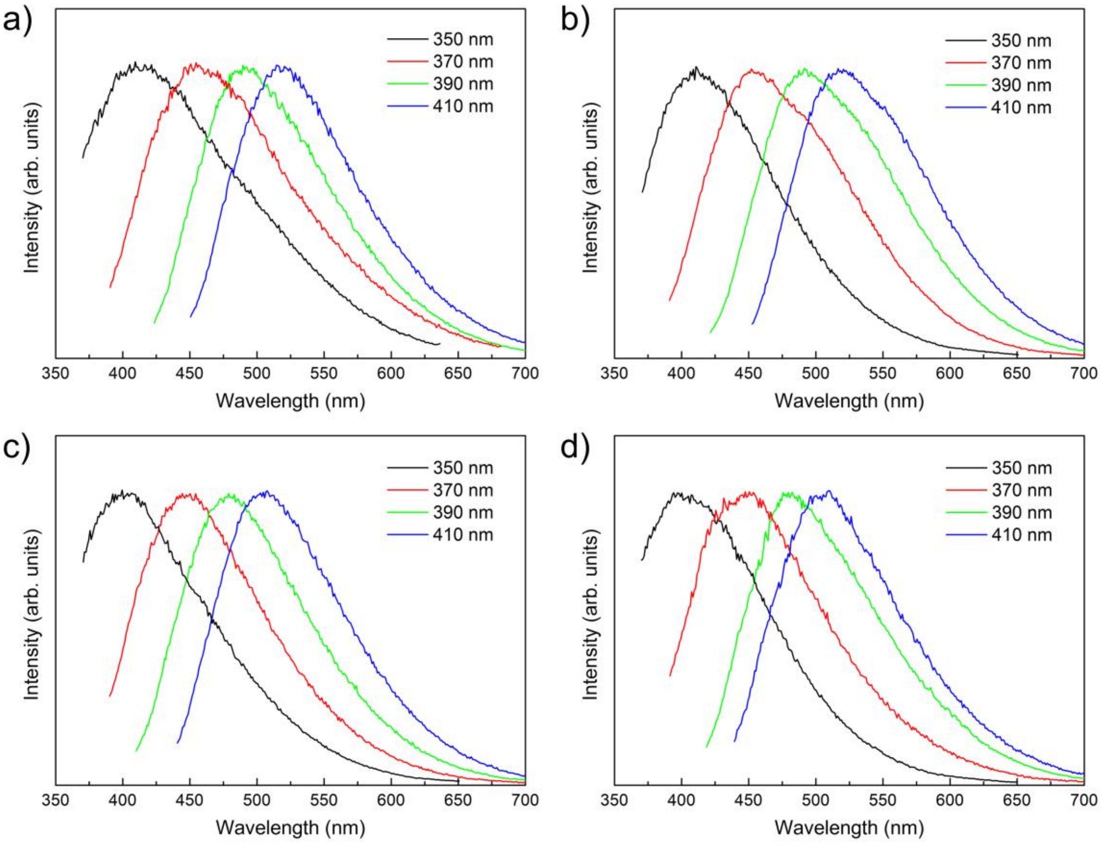

Supplementary Fig. 8. Normalized solid-state emission spectra of a) FLMs (self-assembled from CSE), b) 18RLSs (self-assembled from CNC-C18), c) FMs (self-assembled from CLE), and d) 12RLSs (self-assembled from CNC-C12) at different

excitation wavelengths.

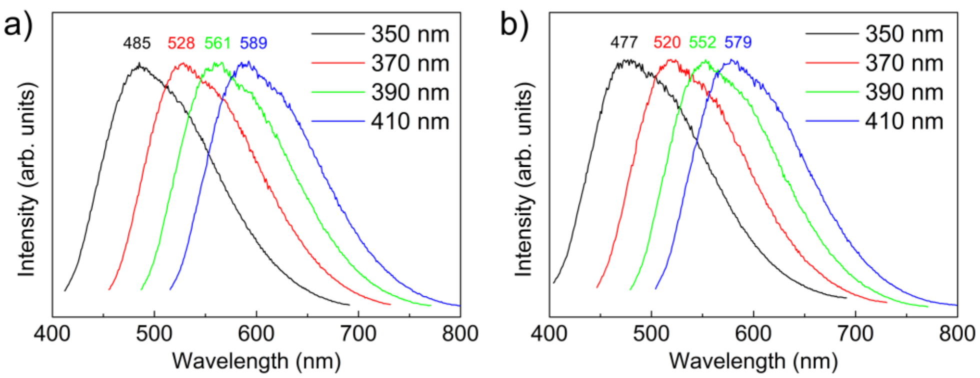

Supplementary Fig. 9. Delayed emission spectra (normalized) of a) MHs and b) BSMPs at different λ_{ex} values at room temperature (t_d = 0.1 ms) (λ_{ex} listed in legend, λ_{max} labelled above the peak).

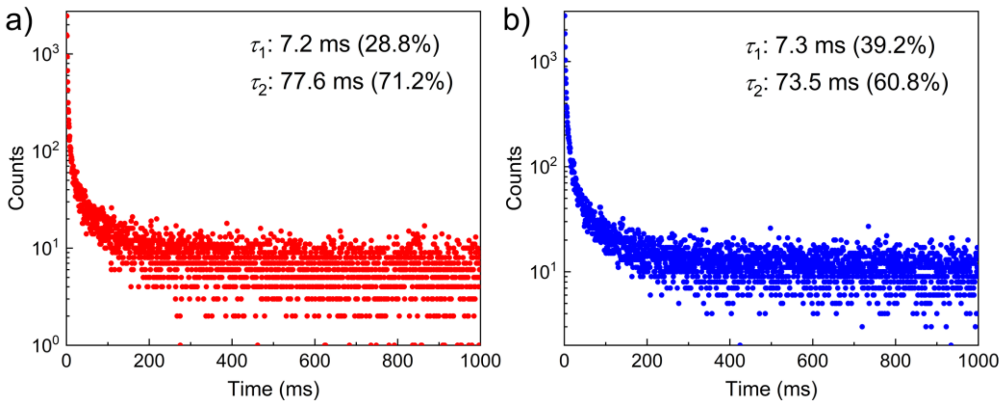

Supplementary Fig. 10. Solid-state phosphorescence lifetimes of a) MHs (λ_{ex} = 350 nm, λ_{em} = 485 nm) and b) BSMPs (λ_{ex} = 350 nm, λ_{em} = 477 nm) at room temperature.

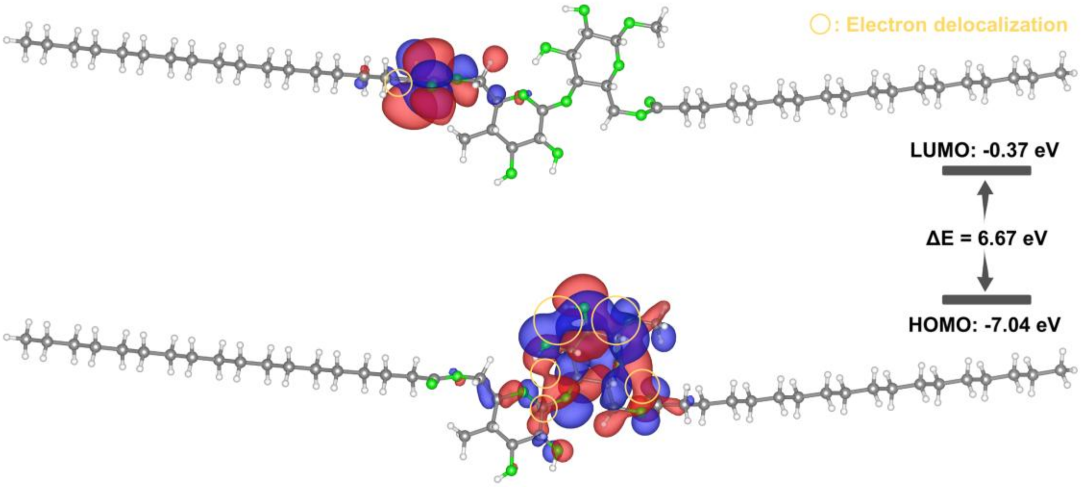

Supplementary Fig. 11. The calculated highest occupied molecular orbitals (HOMOs) and lowest unoccupied molecular orbitals (LUMOs) of 1-C18-DS1 (one repeating unit, modified with stearoyl groups, degree of substitution of 1).

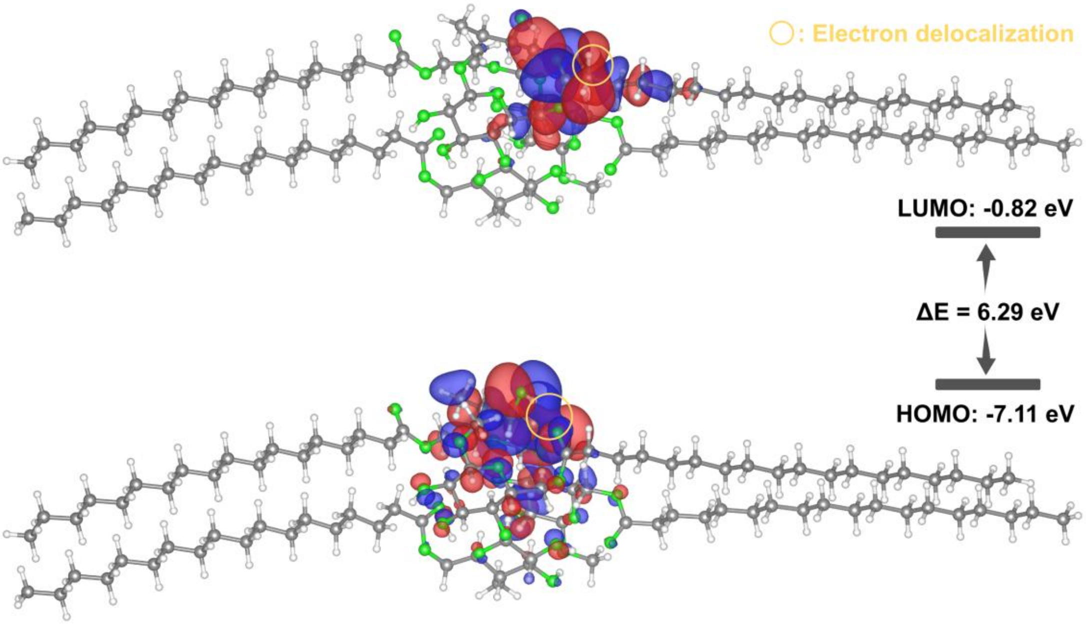

Supplementary Fig. 12. The calculated highest occupied molecular orbitals (HOMOs) and lowest unoccupied molecular orbitals (LUMOs) of 2-C18-DS1 (two repeating units, modified with stearoyl groups, degree of substitution of 1).

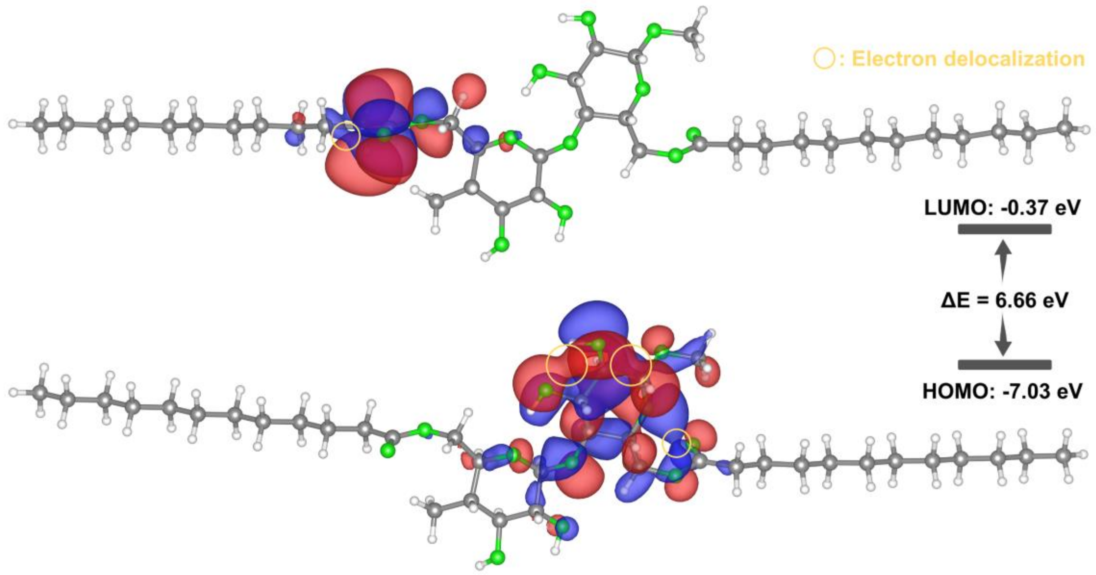

Supplementary Fig. 13. The calculated highest occupied molecular orbitals (HOMOs) and lowest unoccupied molecular orbitals (LUMOs) of 1-C12-DS1 (one repeating unit, modified with lauroyl groups, degree of substitution of 1).

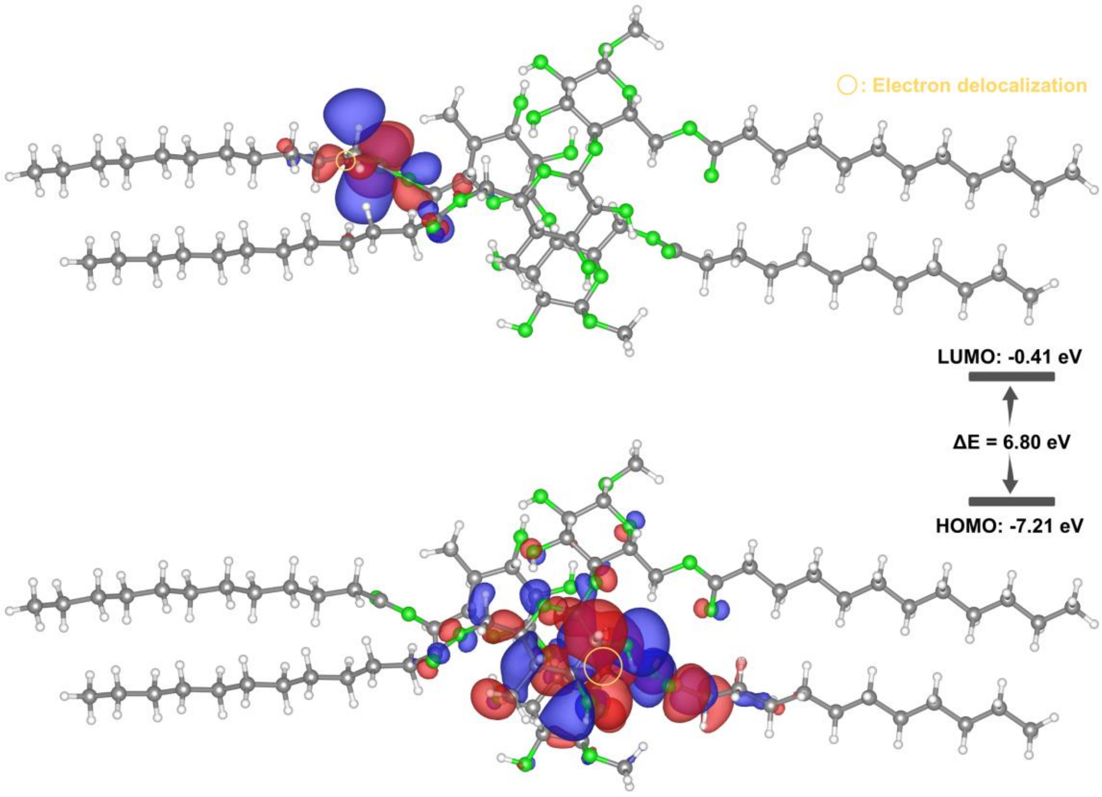

Supplementary Fig. 14. The calculated highest occupied molecular orbitals (HOMOs) and lowest unoccupied molecular orbitals (LUMOs) of 2-C12-DS1 (two repeating units, modified with lauroyl groups, degree of substitution of 1).

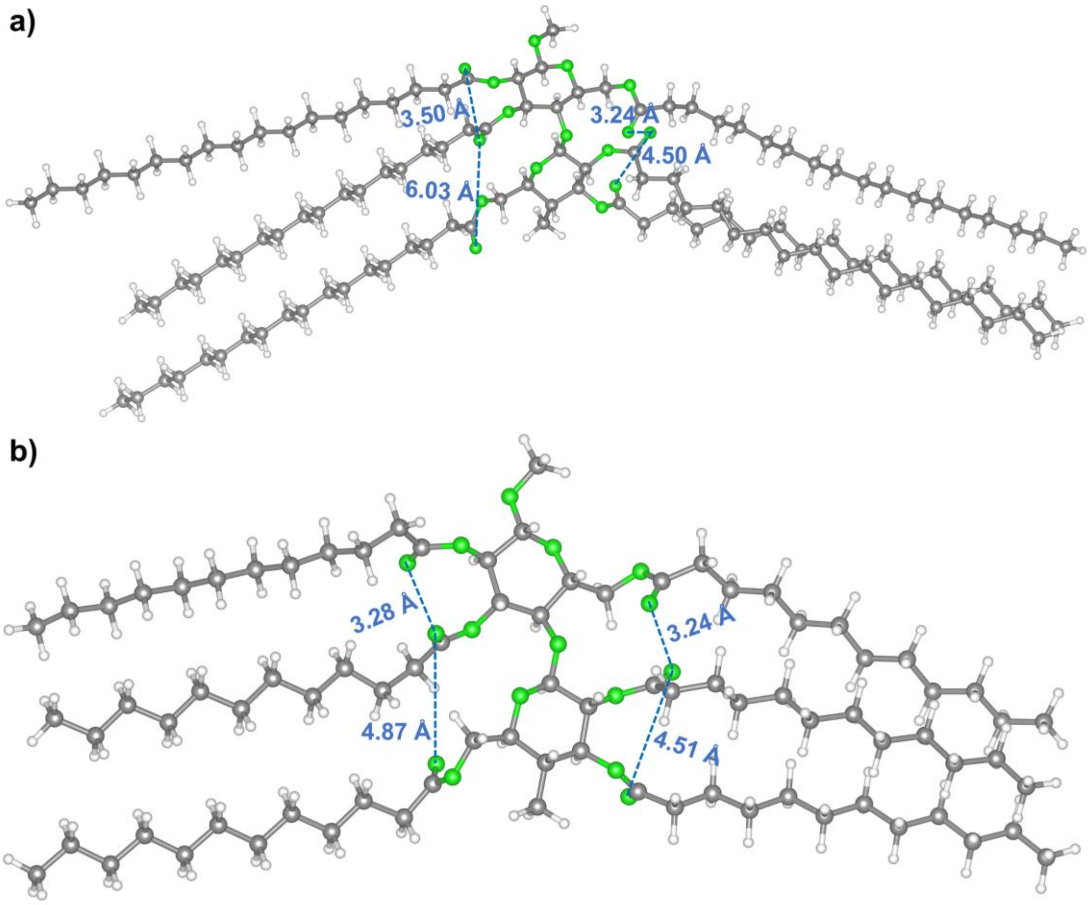

Supplementary Fig. 15. The O-O distances for the ester groups in the DFT-optimized conformations of a) 1-C18-DS3 and b) 1-C12-DS3.

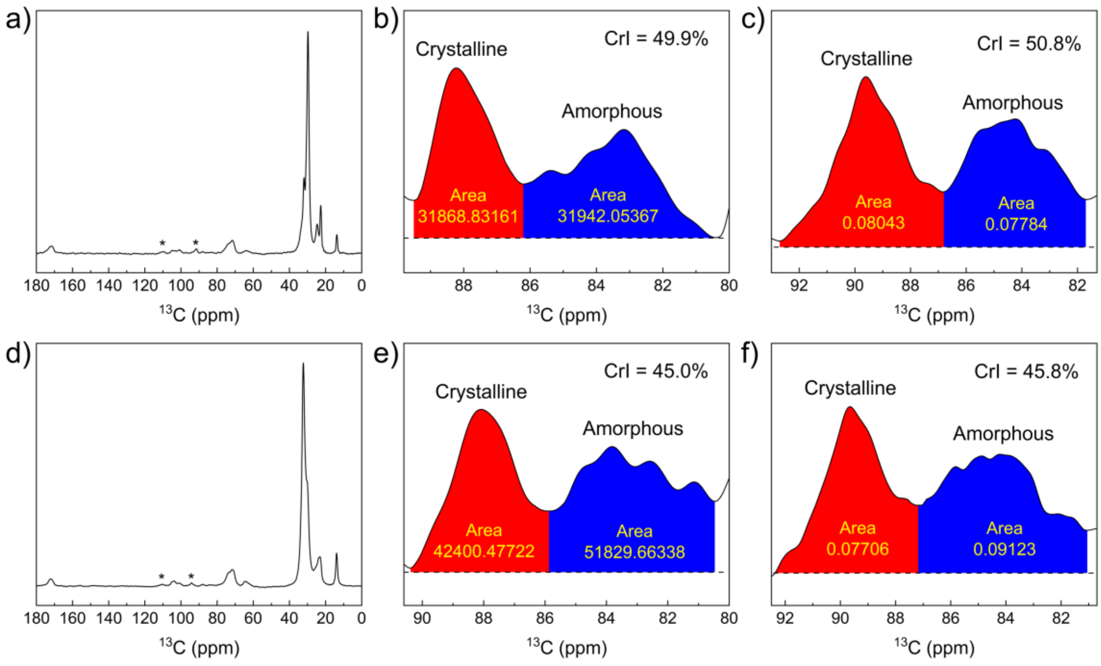

Supplementary Fig. 16. Solid-state ^{13}C NMR spectra of a,b) BSMPs co-assembled from CLE+CNC-C12, c) pristine CNC-C12 prior to assembly (partial enlargement of Supplementary Fig. 2b), d,e) MHs co-assembled from CSE+CNC-C18, f) pristine CNC-C18 prior to assembly (partial enlargement of Supplementary Fig. 2a). The stars label the spinning sidebands. CrI is calculated by dividing the area of the crystalline peak by the total area assigned to the C4 peaks.[2-4]

For cellulose (polymorph I), the CrI remains essentially unchanged when comparing co-assembled CLE+CNC-C12 (BSMPs) with pristine CNC-C12 prior to assembly, as well as co-assembled CSE+CNC-C18 (MHs) with pristine CNC-C18 prior to assembly. Individual CLE/CSE is not considered, as it is fully dissolved in THF before assembly.

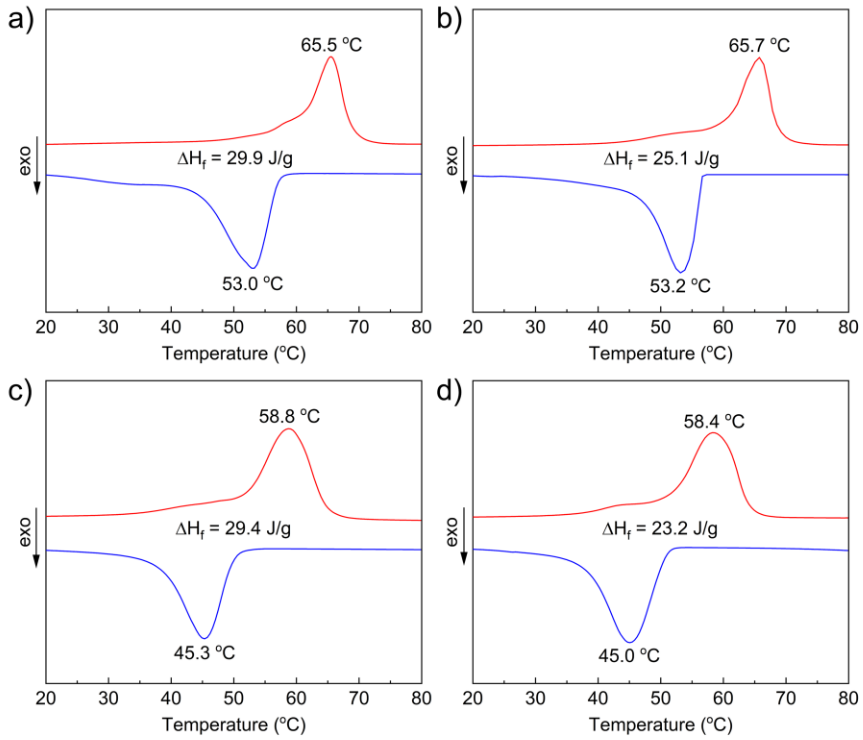

Supplementary Fig. 17. DSC results of a) MHs co-assembled from CSE+CNC-C18, b) pristine CNC-C18 prior to assembly, c) BSMPs co-assembled from CLE+CNC-C12, d) pristine CNC-C12 prior to assembly.

For crystallized fatty-acyl groups, the CrI also remains essentially unchanged when comparing co-assembled CSE+CNC-C18 (MHs) with pristine CNC-C18 prior to

assembly, as well as co-assembled CLE+CNC-C12 (BSMPs) with pristine CNC-C12 prior to assembly (Supplementary Fig. 17, Supplementary Table 2).

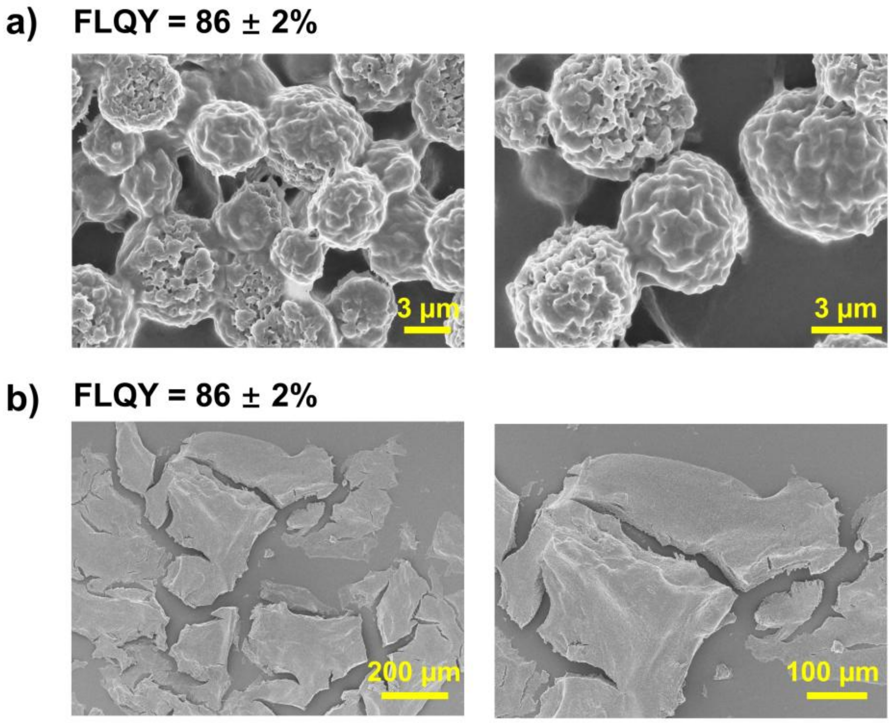

Supplementary Fig. 18. Formation of the coarse microspheres and bulk structures with CNC-C18+CSE after the evaporation of different solvents: a) dichloromethane (DCM), b) trichloromethane (TCM). Their FLQYs were the same as those of MHs.

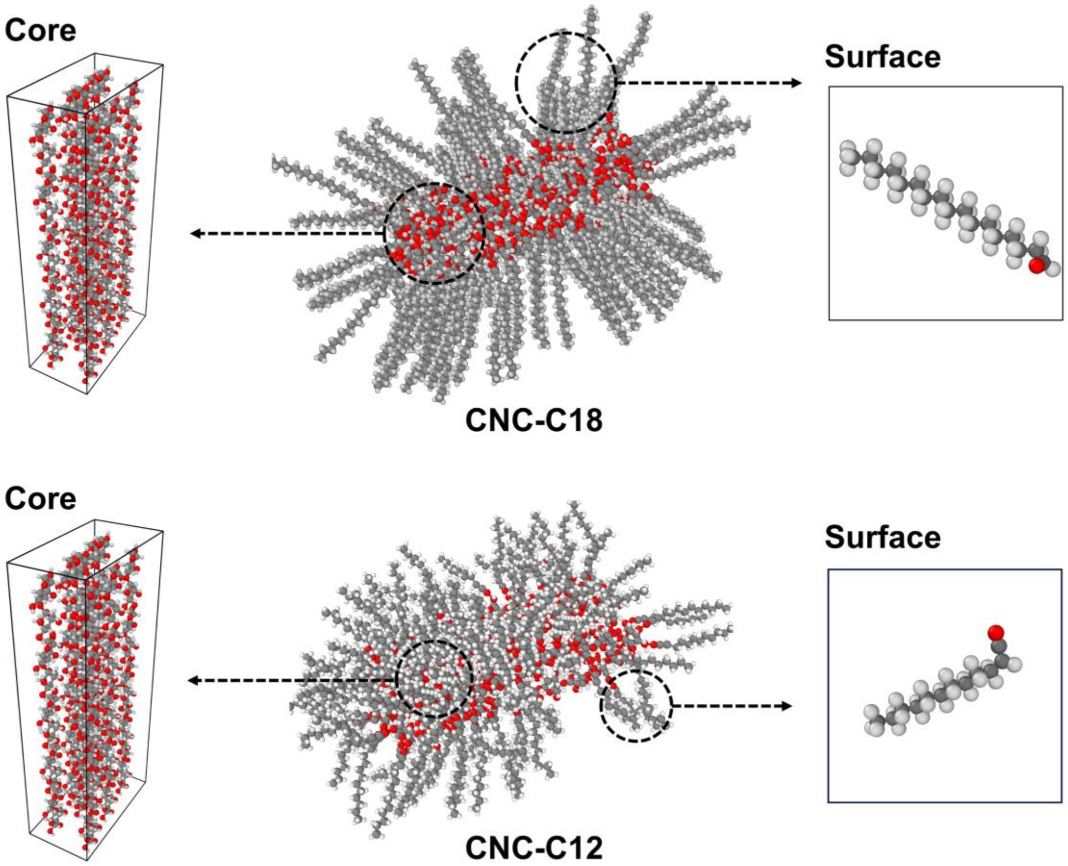

Supplementary Fig. 19. MD modelling of CNC-C18 and CNC-C12, of which the core is CNC with form I crystalline and the surface groups are stearoyl and lauroyl groups.

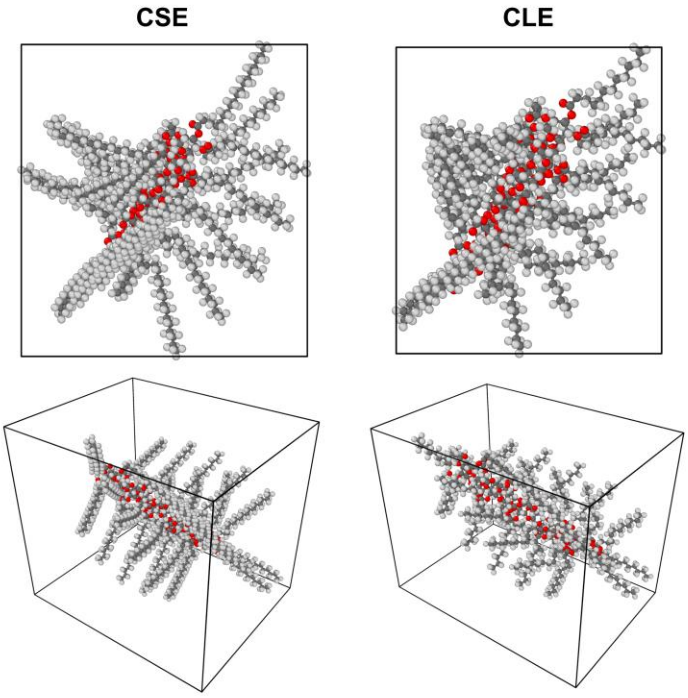

Supplementary Fig. 20. MD modelling of CSE and CLE.

Supplementary Note 1. Calculation of degree of substitution.

DSs were calculated according to the carbon contents, as the following formula:

$$DS = \frac{5.13766 - 11.5592 \cdot C}{0.996863 \cdot C - 0.856277 \cdot n + n \cdot C}$$

where *C* is the content of element carbon and *n* is the number of carbon atoms in the acyl moieties, respectively.

Supplementary Table 1. Degrees of substitution at C2 (DS_2), C3 (DS_3), and C6 (DS_6) calculated from the solid-state NMR spectra.

Sample	DS_2	DS_3	DS_6
CNC-C12	0.26	0.15	0.39
CNC-C18	0.33	0.10	0.43

Supplementary Table 2. CrI values for crystallized fatty-acyl groups, calculated from the DSC results.

Sample	m_{samp} (mg)	ΔH_f (J/g)	m_{crys} (mg)	DS	m_{acyl} (mg)	CrI
MHs	8.9	29.9	1.204	1.51	6.368	18.9%
CNC-C18	9.9	25.1	1.124	0.89	5.902	19.1%
BSMPs	8.7	29.4	1.157	1.49	5.478	21.1%
CNC-C12	9.5	23.2	0.997	0.82	4.582	21.8%

m_{samp}: mass of the sample used for DSC measurement, ΔH_f: enthalpy of heat fusion, m_{crys}: mass of the crystallized fatty-acyl groups, DS: degree of substitution, m_{acyl}: mass of all fatty-acyl groups, CrI: calculated crystallinity index. For sample MHs, mixing equal masses of CNC-C18 and CSE yields an equivalent DS of 1.51, while for sample BSMPs, mixing equal masses of CNC-C12 and CLE yields an equivalent DS of 1.49. CrI = (m_{crys}/m_{acyl}) × 100%.

Supplementary Table 3. Experimental parameters for NMR measurements. For ramped CP, rf-field strengths are calculated at the middle of the ramp.

Sample		CNC-C18	CNC-C12	CSE	CLE	MCC
MAS (kHz)		55.555	55.555	7	8	10
D1 (s)		4	4	10	10	11
Total time (h)		5.8	5.8	20.0	20.0	0.99
CP conditions	^{1}H rf-field strength (kHz)	96.5	96.5	44.75	46	49.5
	^{13}C rf-field strength (kHz)	41.6	41.6	34	36.5	37.5
	Mixing time (ms)	2	2	3	3	1
Decoupling	Type	SW_f-TPPM[5]	SW_f-TPPM[5]	SPINAL64[6]	SPINAL64[6]	SPINAL64[6]
	^{1}H rf-field strength (kHz)	14.4	14.4	83.333	83.333	83.333
	Pulse length (μs)	36	36	5.44	5.44	6
AQ (s)		0.0179	0.0179	0.03	0.03	0.0102

References

1 Takahashi, S.-I., Fujimoto, T., Barua, B. M., Miyamoto, T. & Inagaki, H. 13C-NMR spectral studies on the distribution of substituents in some cellulose derivatives. *J. Polym. Sci. A Polym. Chem.* **24**, 2981–2993 (1986).

2 Salem, K. S. *et al.* Comparison and assessment of methods for cellulose crystallinity determination. *Chem. Soc. Rev.* **52**, 6417–6446 (2023).

3 Liu, P. *et al.* Structure selectivity of alkaline periodate oxidation on lignocellulose for facile isolation of cellulose nanocrystals. *Angew. Chem. Int. Ed.* **59**, 3218–3225 (2020).

4 Park, S., Baker, J. O., Himmel, M. E., Parilla, P. A. & Johnson, D. K. Cellulose crystallinity index: measurement techniques and their impact on interpreting cellulase performance. *Biotechnol. Biofuels.* **3**, 10 (2010).

5 Thakur, R. S., Kurur, N. D. & Madhu, P. K. Swept-frequency two-pulse phase modulation for heteronuclear dipolar decoupling in solid-state NMR. *Chem. Phys. Lett.* **426**, 459–463 (2006).

6 Fung, B. M., Khitrin, A. K. & Ermolaev, K. An improved broadband decoupling sequence for liquid crystals and solids. *J. Magn. Reson.* **142**, 97–101 (2000).
