# Supplementary material for: Self-assembled cellulosic superstructures with unanticipated high quantum yields
Source: Nat Commun. 2025 Dec 10;17:176. doi: 10.1038/s41467-025-66277-8 (PMC12780042; doi:10.1038/s41467-025-66277-8)
Supplement: Supplementary file 2 — Description of Additional Supplementary Files [file 41467_2025_66277_MOESM2_ESM.pdf]

Description of Additional Supplementary Files

Supplementary Data 1: DFT-optimised atomic coordinates for 1-C12-DS1.

Supplementary Data 2: DFT-optimised atomic coordinates for 1-C12-DS3.

Supplementary Data 3: DFT-optimised atomic coordinates for 1-C18-DS1.

Supplementary Data 4: DFT-optimised atomic coordinates for 1-C18-DS3.

Supplementary Data 5: DFT-optimised atomic coordinates for 2-C12-DS1.

Supplementary Data 6: DFT-optimised atomic coordinates for 2-C18-DS1.

Supplementary Data 7: MD-simulated start and end coordinates for the co-assembled system CNC-C18+CSE.

Supplementary Data 8: MD-simulated start and end coordinates for the self-assembled system CSE.

Supplementary Data 9: MD-simulated start and end coordinates for the self-assembled system CNC-C18.

Supplementary Data 10: MD-simulated start and end coordinates for the co-assembled system CNC-C12+CLE.

Supplementary Data 11: MD-simulated start and end coordinates for the self-assembled system CLE.

Supplementary Data 12: MD-simulated start and end coordinates for the self-assembled system CNC-C12.

Supplementary Movie 1: MD-simulated trajectory for the co-assembled system CNC-C18+CSE

Supplementary Movie 2: MD-simulated trajectory for the self-assembled system CSE

Supplementary Movie 3: MD-simulated trajectory for the self-assembled system CNC-C18

Supplementary Movie 4: MD-simulated trajectory for the co-assembled system CNC-C12+CLE

Supplementary Movie 5: MD-simulated trajectory for the self-assembled system CLE

Supplementary Movie 6: MD-simulated trajectory for the self-assembled system CNC-C12
